# Proportions of macronutrients, including specific dietary fats, in prospective anti-Alzheimer’s diet

**DOI:** 10.1038/s41598-019-56687-2

**Published:** 2019-12-27

**Authors:** Marcin Studnicki, Konrad J. Dębski, Dariusz Stępkowski

**Affiliations:** 1 Department of Biometry, Warsaw University of Life Sciences-SGGW, ul. Nowoursynowska 159, 02-776 Warszawa, Poland; 2 Fork Systems, ul. Broniewskiego 10, 05-850 Duchnice, Poland; 3 Laboratory of Molecular Basis of Cell Motility, Nencki Institute of Experimental Biology, ul. Pasteura 3, 02-093 Warszawa, Poland

**Keywords:** Alzheimer's disease, Risk factors

## Abstract

Here we present a novel life-long whole-population study, which enabled us to predict a diet that, in terms of macronutrient proportions, may be prophylactic against Alzheimer’s Disease (AD). The method is based on the existence of oscillations in the correlation between historical per capita personal income (PCPI) and age-adjusted death rates (AADR) for AD for each state of the USA in 2005. These oscillations can be explained by changing proportions of macronutrients in the average American diet between 1929 and 2005. We assumed that reducing future correlation of PCPI with AADR will reduce the population’s susceptibility to AD. Based on the results of fitting macronutrient availabilities to the variability of Roriginal, using Generalized Additive Models (GAM) analysis, we constructed four “Calculator” equations. The Calculator allowed for prediction of an optimal diet characterized by low correlation of PCPI with AADR (Rpredicted) and minimum energy difference from the historical average macronutrient consumption for each corresponding period of life. We predict that protein consumption should be reduced by half in early middle age and late middle age, whereas in late age it should increase. Our predictions are in line with results on humans and simpler organisms in the context of prolonging life.

## Introduction

It is well documented that caloric restrictions (CR) or dietary restrictions (DR) prolong life span across many species (reviewed in^1,2^), it is, however, not so clear whether humans respond to CR or DR in a similar way. Furthermore, the view that, in nutrition, the ratio or balance of macronutrients have strong biological influence is nowadays getting attention^3–5^. Nutrient sensing signaling pathways and their mutual interactions are vigorously studied^6–9^ in search for explanations how the balance of macronutrients may influence health and life span of animals and humans. Specifically, studies on animals fed ad libitum are showing that different ratios of macronutrients are required in a period of reproduction and for prolonging the life span^7,8^. In the period of reproduction high protein diet is preferred, whereas for prolonging life - a low protein diet is more adequate^8^. These observations can probably be translated to human population as the longest living human population on Okinawa consumed a diet characterized by low protein content relative to carbohydrates (mostly complex sugars) and fat (low in saturated fat and rich in omega 3 PUFA fatty acids)^10,11^. It has also been proposed that there are different needs for macronutrient ratios in different periods of life^1,5,12^.

In our previous papers^12,13^ using the “Calculator of anti-Alzheimer’s Diet” developed by us, we have calculated potentially prophylactic proportions of macronutrients (carbohydrates, fat total, and protein, with regard to the presence or absence of alcohol in the diet) in different periods of the human life (youth, early middle age, late middle age and late age). The calculator is based on Alzheimer’s Disease (AD) age-adjusted death rates (AADR) relation to per capita personal income (PCPI) in the USA^14^. The strength of this relation is measured by correlation coefficient R, which is, in turn, proportional to population’s susceptibility to AD^12–14^. We claim that a switch in consumption to the proportions of macronutrients calculated by us, will result in significant reduction of the susceptibility to Alzheimer’s disease and possibly increase the longevity. One of the questions unsolved in previous papers is what the proportions between the intake of different types of fat should be. In the present paper we modified our calculator by implementing Generalized Additive Models (GAM) to calculate optimal proportions between saturated, monounsaturated and polyunsaturated fats as well as protein and carbohydrates. Our results advocate for a significant reduction of protein intake in early middle age and late middle age periods of life in comparison with the average historical consumption. We found also that our predicted values for the intake of specific types of fat do not differ very significantly from the historical consumption.

## Results and Discussion

### Precedence periods and estimation of relative confidence levels of the precedence periods

**Figure 1:**
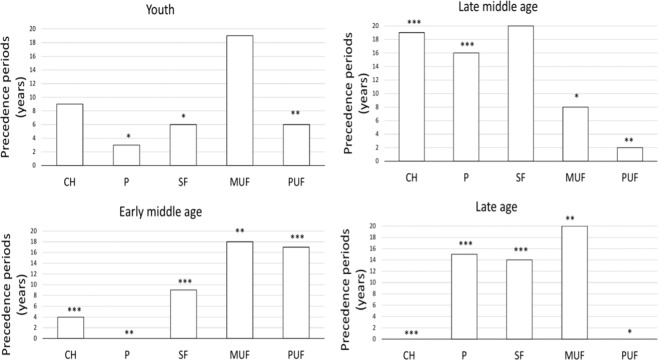
The estimated optimal precedence periods for 5 macronutrients in four periods of life with relative sequence of confidence levels of determination. CH – carbohydrates, P – protein, SF – saturated fat, MUF – monounsaturated fat, PUF – polyunsaturated fat. Three stars – highest confidence, no star – lowest confidence.

The consumed diet exerts its effects, either positive or negative, after a certain period of time, which we call **precedence period**. Using a **global optimization** procedure (see Methods) we determined the **precedence periods** of all nutrients studied for the four **periods of life**. The results are presented in Fig. 1. They vary significantly from 0 till 20 years, depending on the nutrient and the **period of life**. Some have tendency to increase during the life course, as in the case of protein, others to decrease like, for example, PUF. Still others, like carbohydrates, have a U-shaped dependence on time. This variability probably depends on the aging processes occurring during the life course and the differences in metabolic pathways for each nutrient. To determine confidence levels of the estimation of precedence periods we used the method of **relative sequence of confidence** described in Methods. Precedence periods determined for youth have, on average, the lowest confidence for all nutrients. Precedence periods determined for carbohydrates have the highest confidence level (marked with three stars in Fig. 1) in the three last periods of life and the lowest in youth. Precedence periods determined for proteins are characterized by low or high confidence of determination in the two first periods of life, respectively, whereas in the late middle age and late age by the highest confidence. Precedence periods for specific types of fat are determined with, on average, lower confidence levels than for protein and carbohydrates and that change throughout the periods of life.

### Prediction of the proportions of macronutrients

Table 1. presents the results of the “Calculator” output i.e. predicted proportions of macronutrients in percent of energy equivalent and in grams in the diet scaled to 2000 kcal. These data are compared with percent of energy equivalent of mean availabilities and mean availabilities in grams for a given period of life, scaled also to a 2000 kcal diet. This comparison points to the necessity of reducing the protein intake in early middle age and late middle age by half. These predictions are accompanied by high or the highest confidence levels of precedence periods determination. The change is compensated by a slight increase in all remaining nutrients. In the late age we predict higher than historical consumption of proteins whereas in youth the prediction is almost equal to the mean availability in this period of life.

**Table 1:** Comparison of the predicted optimal proportions of macronutrients for prevention of AD, in [%] of energy and in grams per day per capita, scaled to a 2000 kcal diet with mean availabilities scaled also to a 2000 kcal diet in a given period of life.

Macronutrients	Proportions of macronutrients [%]				Grams per day			
	Youth	Early middle age	Late middle age	Late age	Youth	Early middle age	Late middle age	Late age
	Predicted diet							
Carbohydrates	54	54	50	43	269	269	251	218
Proteins	11	7	6	17	57	34	31	84
Saturated Fats	14	17	17	16	33	37	38	35
Monounsaturated Fats	14	16	18	15	30	37	40	33
Polyunsaturated Fats	7	6	9	9	14	14	19	19
Corresponding mean								
Carbohydrates	55	51	48	50	275	254	241	247
Proteins	12	12	13	12	58	60	64	61
Saturated Fats	15	15	14	13	34	35	32	29
Monounsaturated Fats	14	16	17	17	31	35	37	38
Polyunsaturated Fats	4	6	8	8	10	13	17	17

Proportions were calculated as percentage of energy share assuming 9 kcal per gram of fat, 4 kcal per gram of carbohydrates and protein.

### The optimal diet

The suboptimal diet has unexpectedly high influence on the development of non-communicable diseases^15^. Here we present a method of prediction of a life-long optimal diet in regard to macronutrient proportions. The diet human populations are consuming is a complex exposure to many and often synergistic factors. Thousands of bioactive components are present in the diet an individual is consuming^16^. Therefore, exact quantification information concerning the dietary intake is crucial for obtaining reliable results as to the effects of this diet on short term health, health-span and longevity. Nutritional epidemiology relies mainly on observational design of studies, which can be criticized for being dependent on food questionnaires based on the recall of food intake. However, they are usually performed on large cohorts, which reduces bias inevitably present in the food questionnaires. On the other hand, randomized trials are performed on much smaller cohorts and with shorter time of duration^16–18^. Despite all these problems, nutritional epidemiology, backed by short term interventional studies and translational research on animals, provides the background to propose dietary guidelines for the human populations to follow. Taking into consideration the high impact of dietary guidelines on public health, there is an urgent need to provide independent and novel methods of determination of the long term effects of diet on the prevention of AD and other diseases related to old age. Methods that would confirm and complement the results obtained with standard nutritional epidemiology studies. In our previous^12–14^ and the current paper we provide such a new method for predicting the long term effects of a diet based on estimation of the optimal proportions of macronutrient content. Our method could be assigned to the discipline of so called life-course epidemiology^19^.

The main finding of this work is that lower consumption of protein in the mid-life and higher for the period of late age, compared with what was eaten in the USA, may benefit our health and longevity. We propose that these potentially prophylactic proportions of protein to other macronutrients maintained throughout life will reduce the burden of AD and other age related degenerative diseases as well as will extend the health-span and longevity. Our proposal is substantiated by the research on simple organisms and mammals reviewed in^1,2^, as well as on humans^20^. This research has shown, across all the species studied, that protein reduction extends health-span and longevity. In the last period of life - late age, we propose to increase protein consumption above the observed levels. This is consistent with the observations that the overall synthesis of proteins in human organism is compromised at this age^21,22^ (and references therein) due to “anabolic resistance”. Higher protein consumption and subsequent essential amino acids availability from intestines will activate the signaling pathways stimulating protein synthesis, which in turn will improve muscle and overall health. This reasoning is endorsed by the results of Senior *et al*.^23^ who found that at old age mice need higher protein intake than in the early and mid-life. It is interesting to compare the protein/carbohydrates ratio optimal for the lowest mortality and risk of death from AD, for old age mice in^23^ and our results for humans of old age. It is approximately 1:1 for mice and 1:2.5 for humans. In mid-life this ratio is equal to 1:2 for mice and to approximately 1:8 for humans. We predict a ratio of 1:5 as optimal for humans at youth. We predict also that the intake of carbohydrates is in the range of their percentage share in energy, which is consistent with the U-shaped dependence of all-cause mortality and cause-specific mortality on carbohydrates consumption^24^. Our results correspond to the optimum for the lowest mortality^24^.

Wang *et al*.^25^ reported associations between specific types of fat intake and total mortality. They found positive associations of increased intake of saturated fat with total mortality, whereas increase in the intake of monounsaturated and polyunsaturated fats was negatively associated with mortality. Our predictions rank within the highest quintiles in Wang *et al*. or slightly above for all types of fats. We think that the potential influence on mortality of increased saturated fat intake we predict is counterbalanced by increased intake of unsaturated fats.

In the present paper we used de novo global optimization of five macronutrients whereas in the previous paper^13^ the global optimization was performed on three macronutrients (total fat instead of specific types of fats). It is interesting that comparison of the results for predicted carbohydrates and protein content are similar, which strengthens the reliability of our results.

### Limitations of our studies

The crucial assumption we made is that the oscillations of Roriginal in time can be explained by the variability of macronutrient consumption by the population of the USA. The only proof of this concept are the rational results obtained as the output of the “Calculator” e.g. reasonable predictions of prophylactic diet supported by the results of other studies^1,2,6,7,20,23,24,26,27^. Besides that, the main limitation of our studies are the input data of macronutrient availabilities. The possible bias related to the values we have used (SI Dataset S1) is difficult to judge. For most of the macronutrients we used a 5 g step in our calculator, which seems to be a reasonable estimation of the errors of data presented in Table 1. We have assumed that the consumption within the population follows the normal Gaussian distribution and the average consumption per-capita covers the majority of population and all age groups and both sexes. We did not consider the medical conditions of the population since, it would be extremely difficult, if not impossible, to do it for the whole population data for the USA. We assume that our predictions are valid for the healthy part of the population. We also did not differentiate the source of protein i.e. animal or plant due to lack of such data available to the public. This may influence the results as consumption of plant protein instead of animal protein is considered as more healthy^28^. Due to the nature of our analysis it is not possible to introduce exact age ranges for the four periods of life. This is because in the 2005 the population of people dying from AD was of different age. We do not have data about their average age. In this case we think that it is better to leave age limits as they are generally thought to be and not to assign strict boundaries. Youth poses a special problem with assigning boundaries since it is not clear from our studies when it begins. It might be even childhood. The other source of bias is the relation between availability per capita and average consumption per capita, which is unknown. We assumed that the availability is proportional to average per-capita consumption. Since we have obtained a reasonable diet content in regard to consumption of macronutrients, which is backed up by studies done by others^1,2,6,7,20,23,24,26,27^, these assumptions are most probably valid. The message described in this paper is important for Public Health and we hope it will prompt new research by standard epidemiological methods to confirm it. Appropriate measures should be then applied to deliver this message to the general public.

## Conclusions

We predict that the proportions of macronutrients in the diet in four periods of life (youth, early middle age, late middle age, late age) should differ, especially in regard to protein intake. Specifically, we postulate that protein intake in early middle age and late middle age should be reduced by half by comparison to historical consumption in the USA. Conversely, in the late age, the protein intake should be increased above the historical consumption. We postulate that applying these changes in the consumption of protein throughout the whole life of an individual will act prophylactically against development of AD and possibly will extend health-span and the duration of life

## Methods

**Figure 2:**
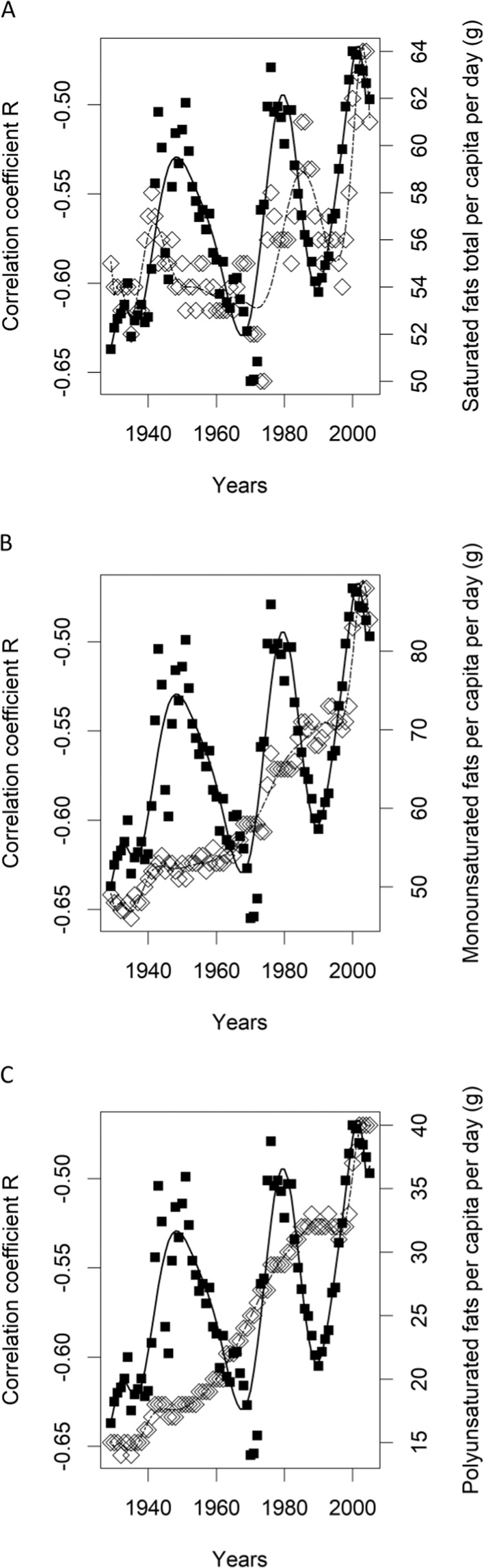
The variability of Roriginal^13^ and time course of the availabilities of specific types of fat (g per day per-capita) in the period 1929–2005. For numerical data see SI Dataset S1 (**A**) saturated fat, (**B**) monounsaturated fat, (**C**) polyunsaturated fat. The time courses of availabilities of total fat, carbohydrates and protein versus Roriginal are presented in the paper by Studnicki *et al*.^13^. Filled squares - Roriginal, empty rhombi -specific types of fat.

The graphical representation of input data variability in time is presented in Fig. 2

The flow charts of the “Calculator”

Flow chart 1. General procedure.

Flow chart 2. Global optimization procedure.



Flow chart 3. Procedure of calculation of optimal diet in regard to macronutrients proportions.



The calculator from our previous papers^12,13^ was modified and optimized to obtain precedence periods for carbohydrates, protein, saturated, monounsaturated, and polyunsaturated fat. We used global de novo optimization for all 5 nutrients within GAM analysis procedure instead of multiple regression. This resulted in 4084101 variants of sets of precedence periods when using back steps up to −20 years. From them the one variant with the highest R was chosen as the optimal one (**Roptimal**). The results from GAM analysis with **Roptimal** for each period studied were chosen as calculator equations and are presented in SI Dataset S2. We obtained four equations corresponding to four periods of life: **youth, early middle age**, **late middle age** and **late age**. These equations were then used to generate a set of tables with new proportions of macronutrients to minimize **Rpredicted** and optimal proportions (optimal diet) were chosen as in our previous papers^12,13^ except that the corresponding mean availability was calculated without back shift by precedence periods. The codes in R program for optimization, calculation of Rpredicted and minimum energy difference together with the manual are available in a public repositories at https://bitbucket.org/seventm/fsproj_alzheimeroptimaldiet and https://github.com/seventm/fsproj_alzheimeroptimaldiet/releases/tag/v1.0, 10.5281/zenodo.3574598. The computations were performed on the computer cluster with 96 cores and 1TB of RAM. Parallel computing approaches based on *snow* packages are implemented in our R codes. The approximate time used for calculating results of optimization for one period was around 10 minutes.

### GAM analysis

Calculation of the optimal diet was based on a linear relationship between Roriginal (as predicted variable) and macronutrients (in grams of daily availabilities per capita) with separate types of fats (as predictor variable) modelled using Generalized Additive Models (GAM). In contrast to multiple regression model the GAMs are not assuming the linearity and normality of the error distribution. The GAMs are employed by modeling the expected value of Roriginal as follows:

$$\begin{array}{rcl}{\rm{Roriginal}} & = & \alpha +{{\rm{f}}}_{1}({\rm{carbohydrates}})+{{\rm{f}}}_{2}({\rm{protein}})\\  &  & +\,{{\rm{f}}}_{3}({\rm{saturated}}\,{\rm{fat}})+{{\rm{f}}}_{4}({\rm{monounsaturated}}\,{\rm{fat}})\\  &  & +\,{{\rm{f}}}_{5}({\rm{polyunsaturated}}\,{\rm{fat}})\end{array}$$

where: α is an intercept; f_1_,f_2_,f_3_,f_4_ and f_5_ are unknown smooth *nonparametric* functions. In order to obtain an inference on and composition of a new diet more easily, we used linearized smooth functions described in^29^. The GAM was fitted using the function *gam* from package *mgcv* in the R environment (version 3.5.1; R Development Core Team, 2019)^30^.

### Determination of confidence levels of precedence periods estimated from global optimization

Global optimization was performed to find a set of **optimal precedence periods** corresponding to maximal R (**Roptimal**) for all five nutrients. **Roptimal** was calculated from regression procedure, implementing Generalized Additive Models as described above, using back shift of nutrients availability up to −20 years with a step equal to one year. For each shift for a given nutrient (starting from 0 to −20 years) R coefficients corresponding to all combinations of shifts for other nutrients were calculated. For each nutrient we obtained 21 sets of R. Subsequently **Rmean** and standard deviation (**SD**) **of Rmean** for each set were determined. Precedence periods corresponding to **Roptimal**, maximum **Rmean** and minimum **SD of R mean**, respectively, were used to calculate the **mean precedence period** and **SD of the mean precedence period**. The **optimal precedence periods** and **SD of the mean precedence periods** applied to them are presented in Fig. 1. The SD of the mean precedence period was divided into ranges corresponding to the highest confidence (from 0 to 2; ***), high confidence (from 2 to 5; **), lower confidence (from 5 to 8; *), and the lowest confidence (above 8;). The ranges are determined arbitrarily to give a relative sequence of confidence levels of the determined precedence periods. The procedure described above assumes that all three criteria of determination of the precedence periods: maximum R (**Roptimal**), maximum **Rmean**, minimum **SD of Rmean** are equally important. However, we postulate that the first criterion, **Roptimal**, which represents the best fit of macronutrients to **Roriginal** variability, is the strongest one and the other two are rather auxiliary criteria, which help to get some inference about the confidence with which the precedence periods are determined. Therefore, the **SD of** the **mean precedence period** presented in SI Dataset S3 is overestimated.

**The nomenclature used in the present paper:**

**Roriginal** – Correlation coefficients from the paper by Stępkowski *et al*.^14^ corrected in^13^. Denotes correlation between PCPI (per-capita personal income) from 1929–2005 and 2005 AADR (age-adjusted death rates) of Alzheimer’s Disease (AD) for each state of the USA.

**Roptimal** – The maximal R obtained from GAM analysis using global optimization of precedence periods.

**Rpredicted** - The correlation coefficient calculated from calculator equation corresponding to nutrient coefficients derived from global optimization using discrete steps of the amounts of all nutrients.

**Rmean** – mean of all R calculated from global optimization for a given nutrient and a given period of precedence for it in one of the periods of life.

**SD of Rmean** – standard deviation for Rmean.

**GAM** – Generalized Additive Models.

**Energy difference** – Difference between energy equivalents of mean diet in a given period and predicted diet calculated as a sum of differences for each nutrient. Assuming 4 kcal for 1 g of carbohydrates and protein and 9 kcal for 1 g of fat.

**Global optimization** – GAM analysis of all combinations of precedence periods for all nutrients starting from 0, −1, −2 ……….. up to −20 years. It gives 4084101 combinations of sets of precedence periods.

**Optimal predicted diet** – diet corresponding to minimum energy difference.

**Precedence periods** – periods with back steps equal to one year from 0 up to −20 years.

**Mean precedence period** – mean period of precedence derived from periods estimated by three criteria: optimal precedence period for a given nutrient – corresponding to Roptimal, maximum Rmean, minimum of SD of Rmean.

**SD of mean precedence period** – standard deviation of mean precedence period.

**Optimal precedence period for a given nutrient** – precedence period corresponding to Roptimal.

**Periods of life: youth** - corresponds to the period 1929–1949; **early middle age** – corresponds to the period 1949–1970; **late middle age** - corresponds to the period 1970–1990; **late age** - corresponds to the period 1990–2005.

**Relative sequence of confidence** – The sequence of confidence levels with which the precedence periods are determined as described in chapter “Determination of confidence levels…..”.

## Supplementary information

Supplementary Information

Supplementary Datasets 1–3.

## Data Availability

The input data and results of calculations are available in the Supplementary Information files. The software written for the purpose of calculations employed in this project is deposited at public repositories https://bitbucket.org/seventm/fsproj_alzheimeroptimaldiet and https://github.com/seventm/fsproj_alzheimeroptimaldiet/releases/tag/v1.0, 10.5281/zenodo.3574598. All other data are available from authors on request.
