# Supplementary material for: Proportions of macronutrients, including specific dietary fats, in prospective anti-Alzheimer’s diet
Source: Sci Rep. 2019 Dec 27;9:20143. doi: 10.1038/s41598-019-56687-2 (PMC6934814; doi:10.1038/s41598-019-56687-2)
Supplement: Supplementary file 1 — Supplementary Information [file 41598_2019_56687_MOESM1_ESM.docx]

Supplementary Information for

**Proportions of macronutrients, including specific dietary fats, in anti-Alzheimer’s and pro-longevity diet**

Marcin Studnicki, Konrad J. Dębski, Dariusz Stępkowski

Dariusz Stępkowski

Email: d.stepkowski@nencki.edu.pl

**This PDF file includes:**

Supplementary Information Text

Captions for Datasets S1 to S3

**Other supplementary materials for this manuscript include the following:**

Excel spreadsheet with Datasets S1 to S3

Supplementary Information Text

The codes in R program for optimization, calculation of Rpredicted and minimum energy difference together with a manual are available in a public repository at <https://bitbucket.org/seventm/fsproj_alzheimeroptimaldiet>. (deposited on 24 May 2019).

Dataset S1

Contains the following data: Roriginal from the paper by Studnicki et al. ^13^; macronutrient availabilities g/day per capita. The table of macronutrient availabilities is adapted originally from Table published online by USDA. Source: Calculated by USDA/Center for Nutrition Policy and Promotion. Data last updated Feb. 1, 2015).

[https://www.ers.usda.gov/data-products/food-availability-per-capita-data-system/food-availability-per-capita-data-system/#Archived%20Nutrient%20Availability%20Table](https://www.ers.usda.gov/data-products/food-availability-per-capita-data-system/food-availability-per-capita-data-system/#Archived Nutrient Availability Table)

**Dataset S2**

Contains the results of the best fit of macronutrient availabilities to Roriginal variability. Results obtained by implementing GAM analysis as described in the Methods.

Dataset S3

Contains the results of the analysis of relative sequence of confidence levels of the estimation of precedence periods.
